# 3D-Printed Models for Surgical Planning in Complex Congenital Heart Diseases: A Systematic Review

**DOI:** 10.3389/fped.2019.00023

**Published:** 2019-02-11

**Authors:** Clément Batteux, Moussa A. Haidar, Damien Bonnet

**Affiliations:** Department of Congenital and Pediatric Cardiology, Centre de Référence Malformations Cardiaques Congénitales Complexes, Hôpital Necker-Enfants Malades, Assistance Publique-Hopitaux de Paris, Université Paris Descartes, Sorbonne Paris Cité, Paris, France

**Keywords:** congenital heart disease, 3D technology, segmentation, printed models, accuracy, reliability, surgical planning

## Abstract

**Background:** 3D technology support is an emerging technology in the field of congenital heart diseases (CHD). The goals of 3D printings or models is mainly a better analysis of complex anatomies to optimize the surgical repair or intervention planning.

**Method:** We performed a systematic review to evaluate the accuracy and reliability of CHD modelization and 3D printing, as well as the proof of concept of the benefit of 3D printing in planning interventions.

**Results:** Correlation studies showed good results with anatomical measurements. This technique can therefore be considered reliable with the limit of the operator's subjectivity in modelizing the defect. In cases series, the benefits of the 3D technology have been shown for describing the vessels anatomy and guiding the surgical approach. For intra-cardiac complex anatomy, 3D models have been shown helpful for the planification of intracardiac repair. However, there is still lack of evidence based approach for the usefulness of 3D models in CHD in changing outcomes after surgery or interventional procedures due to the difficulty to design a prospective study with comprehensive and clinically meaningful end-points.

**Conclusion:** 3D technology can be used to improve the understanding of anatomy of complex CHD and to guide surgical strategy. However, there is a need to design clinical studies to identify the place of this approach in the current clinical practice.

## Introduction

Benefits of 3D technology in refinement of the anatomic diagnosis of congenital heart diseases (CHD) are increasingly reported. The different facets of this technology are helpful in the setting of the large anatomic variation and complexity of CHD. Hitherto, however, the usefulness of 3D technologies to fine-tune surgical or catheter-based procedures has to be explored. It is now possible to build a 3D laboratory in a tertiary surgical center, and to manage locally the process from the treatment of a digital imaging and communication in medicine (DICOM) file to the final obtaining of a 3D printed model.

The sequential process for obtaining 3D models that can be shared for communication can be summarized in original acquisition, images segmentation, post-processing, and printing (1–3). Orthopedics and maxillofacial have been pioneers in this field.

## Method

Different studies suggested that 3D could improve medical understanding (4, 5), teaching (6–8), surgical training (9, 10) and patient information (11) in CHD. Still, there is no clear demonstration of these benefits in well-designed studies. Notwithstanding this lack of data, it is highly probable that the decreasing cost of printed 3D models of CHD (12) will modify the current approach of CHD in a short future. Intuitively, these models will help to share complex information from different imaging modalities between caregivers. Convenience of these 3D models has to be clearly evaluated, as congenital cardiologists have to be trained to manipulate these information and to understand the limits of the different steps in the process.

The next phase is to demonstrate the value of these 3D models in planning surgical procedures in complex CHD. Certainly, there should be a proof of concept step before designing trials aiming to show the efficiency of these models in improving outcomes.

Here, we reviewed the available literature on intervention planning in complex CHD with the help of 3D models. Our aim was to set up the bases for a study aiming to demonstrate the benefit of these models in modifying outcomes after surgical or interventional procedures.

We performed an extensive literature's search using PubMed and Google Scholar. At least 3 of 4 the following terms were combined in the query: 3D printing, 3D printed model, complex congenital heart disease, and surgery. The terms surgical planning, and measurement correlation were optionally added into the query. Two congenital heart diseases specialists reviewed all retrieved abstracts. Publications describing only medical comprehension of CHD and/or patient information with 3D-models were excluded. All articles describing surgical strategy or measurement's correlation in CHD were included and analyzed.

## Results

### Accuracy and Reliability of a 3D Printed Model of Congenital Heart Diseases

The first step for this new technology was to validate the reproducibility of 3D models with the original source by measurement's accuracy studies.

The analysis of the different processes to obtain a 3D printed model is necessary to understand that 3D segmentation and printing open the way to possible measurement's bias. The potential risk that the final 3D printed model would not be anatomically similar to the original should indeed be carefully estimated.

#### Pre-segmentation and Segmentation Processes

To create a 3D printed heart, the first step is to proceed to the segmentation of a model from various imaging modalities (cardiothoracic computed tomography CT, Magnetic Resonance Imaging MRI, Transthoracic Echocardiography TEE). Segmentation means treating DICOM's images from a stack of slices to a 3 dimensional scene. To do this, each structure of a scene is isolated as a model and reported in the 3D field by a segmentation process, as for example: blood pool, myocardium, great arteries, coronary arteries. Pre-segmentation consists in isolating a chosen structure, by using an algorithmic treatment of pixels: tresholding, edge attraction, clustering, classification, are the four more used pre-segmentation modes.

Then, segmentation consists in filling the isolated structure in the 3-dimensions fields; then the structure is transformed from slices of pixels to a 3 dimensional model with proper X;Y;Z coordinates. To proceed in a segmentation, a semi-automatic mode is often used. The presegmentation mode can be modified and the segmentation filling is guided by the operator who defines which structure have to be segmented and then let the algorithm work to complete the process.

#### Different Segmentation Softwares and Printing Modality

A large variety of segmentation softwares are used. Most of them involve a human intervention to obtain a final 3D model. This is clearly a source of uncertainty. Total 3D-coordinates of a model is called a mesh, and the most frequently used computed-format to save this model is the Standard Tessellation Language (STL), that will be used for the final printing.

This final step of printing adds another source of discordance between the original source and the final printed piece. These discordances are closely dependent on the type of printer and on the modality for printing (FDM: fused deposition modeling, stereolithography, SLS: selective laser sintering, polyjet). The reliability of the final printed model depends on a multisteps process in which almost each phase creates additive errors and/or approximations.

#### Choice of Segmentation Software

The choice of segmentation software depends on the expectations of the user as well as on his confidence in 3D segmentation and modeling.

Two different categories of 3D modelers can be identified. The first category of users usually work with a turn-key product predefined and automatic segmentation, and including all processes permitting to obtain the final 3D model within a single software. This easy-to-use solution, as proposed by Materialize (Mimics), represents a quick and easy way to perform 3D modeling in medicine. The limits of this type of practice are the cost of the license (more than 10,000$ per year) and the lack of independence for the user during the 3D modeling, the post-processing, and the pre-printing step.

Conversely, the second group is that of self-taught users. Using open-source softwares (Itk-snap, slicer), they can control each step of the segmentation by manually modifying the primary DICOM contrast or the threshold. They can either easily manage the propagation of the segmentation and the post-processing steps. As these software are open-source, they can be downloaded for free and they are frequently upgraded by the developers. The main limit of this approach is the need for a learning curve and the risk of potential subjective bias in the modeling.

#### Correlation Studies

Correlation studies evaluate the concordance between a printed model and the original structure (13–23) (see details in Table 1).

**Table 1:** Measurement's accuracy with 3D printed models.

**Authors**	**Case**	**Comparison**	**Software**	**Structure**	**Measure (model × structure × operator)**	**Pearson correlation**	**Mean diff (mm)**	**Analysis**
Lau et al. (13)	1	3D vs. CT	Mimics	VSD/Aorta/PB	1 × 3 × 2	0.99	0.23	Pearson
Valverde et al. (14)	20	3D vs. CT	Itk-snap	10 vascular diameters	320	0.99	0.27	Pearson
Greil et al. (15)	1	3D vs. mortem	Visualization toolkit	Distances	unclear		0.27	Interactive closest point
Ma et al. (16)	35	3D vs. vivo	Philips EBW	VSD	35 × 1 × 1		No diff (*t* = 0.83, *P* = 0.412 > 0.05)	*t*
Olejnik et al. (17)	8	3 D vs. vivo	3D slicer	Vascular diameters and distance	8 × 3 × 1		0.13 ± 0.26	Bland-Altman
		3D vs. dicom			8 × 5 × 1		0.19 ± 0.38	
Valverde et al. (18)	1	3D vs. MRI	Ayra	Aorta diameters	2 × 8 × 2		0.18 ± 0.38	Pearson and Bland-Altman
		3D vs. X ray			2 × 8 × 2		0.55 ± 0.46	
		Global					0.36 ± 0.45	
Zhao et al. (19)	8	3D vs. CT	Mimics	VSD/Ao arch/PA	8 × 3 × 1	0.977		Pearson
Olivieri et al. (20)	9	3D vs. echo	Mimics	VSD and perivalvar leak	22	0.988	0.4 ± 0.9; t : NS	Pearson Bland-Altman and t
Ngan et al. (21)	6	3D vs. CT	Mimics	MAPCA diameters	6 × 1 × 1		0.26–0.85	*t*
							97% accuracy feeling	
Farooqi et al. (22)	19	3DM vs. 3DBP	Mimics	GA/IVC/SVC/PV	19 × 5 × 4 × 2		Blood pool > myocardium	Anova
Farooqi et al. (23)	6	3D vs. MR	Mimics	AA/VSD/RVLA	6 × 3 × 1	0.99		Pearson
						AA 0.88 VSD 0.74 RV 0.99		

*CT, computed tomography; MRI, Magnetic Resonance Imaging; 3DM, 3D myocardium; 3DBP, 3D bloodpool; VSD, ventricular septal defect; PB, pulmonary branch; Ao Arch, aortic arch; MAPCA, major aortopulmonary collateral artery; SVC, superior vena cava; IVC, inferior vena cava; Ao, Aorta; PA, pulmonary artery; PV, pulmonary valve; AA, aortic annulus; RVLA, right ventricle long axis*.

The methods of these 11 studies were variable, using either an original substrate or DICOM sliced usual imaging modalities (13, 14, 17–23) (TEE, CT, MRI), or angiography (18), per-operative (16, 17) or postmortem (15) measurement as controls. The techniques for measuring the 3D printed model measurement were not homogeneous: directly on the printed model or indirectly from a CT of the printed model. The number of operators, the structures, and the number of measurements were highly variable. Despite this methodological heterogeneity, the correlation between the measurements made with the 3D printed model and the original structure was always satisfactory. Therefore, confidence should be given to the process of segmentation and printing at least for the size of the cardiac structures. It seems reasonable to evaluate this technology in surgical planning.

For the segmentation of the myocardium, two methods have been compared by Farooqi et al. (22): the first direct method used a presegmentation algorithm to isolate the myocardium, this region was then filled; the second indirect method identifies the bloodpool, and then create a shell with an arbitrary thickness surrounding the bloodpool surface to represent the myocardium. In this latter method, the inner surface represents the endocardial surface anatomy, whereas the outer surface is a fabricated structure.

Whereas, the different methods used to analyze the accuracy of 3D models are heterogeneous, they show an acceptable correlation between the 3-D printed-model and the “original structure” with differences being always infra-millimetric. These data suggest that 3D-models could be reliable to appreciate the complex anatomy of cardiac structures in CHD and to adequately plan surgical interventions.

### Surgical Planning With 3D Printed Models—Proof of Concepts by Cases Reports and Single-Center Experiences

The number of case reports suggesting that the 3D visualization and/or printing of complex CHD are helpful in planning surgery or interventional procedure is increasing every day. To summarize, the 3D printed models have been used with two aims.

The first group of case reports describes the anatomy of extra-pericardial structures (great vessels, their branches, intra-thoracic course and relationship with other organs such as the trachea and esophagus) with the objective to refine the diagnosis and to precise the surgical repair (see Table 2) (4–32).

**Table 2:** Cases reports about extra-pericardial benefits of 3D-printed models in congenital heart diseases' surgical planning.

**References**	**CHD**	**Problematic?**	**Benefit of 3D printed model?**
**VESSELS VIEWING**			
Biglino et al. (24)	Truncus arteriosus	Aorta-RPA relationship?	Better surgical approach, need of CPB
Deferm et al. (25)	TOF	Pulmonary arteries?	Better understanding of distal circulation
			Relationship with bronchal tree
Sodian et al. (26)	Komerell with ROLSCA	Anatomic complexity of aortic arch	Intra-operative view to guide surgery
Ryan et al. (27)	TOF	MAPCA localization	Sustend to cath to isolate a MAPCA
Anwar et al. (28)	Vasc ring, TOF, TAPVR	Vessels disposition and diameters and relationship	Surgical viewing and approach
	Heterotaxy with 1V, DTGA Senning		Direct observation in the operating room
		Pulmonary veins approach	Show anatomy and spatial relationship
Ngan et al. (21)	PAVSD	MAPCA?	Peroperative Visualization number of MAPCA
Schmauss et al. (29)	Heart transplantation	Complex vessels anatomy	Surgical planning by understanding anatomy
	ROSCA	Complex surgical approach	Perioperative orientation by conditionnement
**HEART TRANSPLANTATION**			
Smith et al. (30)	Situs in vs.-DORV-TGA going to HTx	Complex anatomy	Understanding anatomy
			Upgrade surgical approach
			Reduce CPB time
Sodian et al. (31)	Failing 1V repair going to HTx	Complex vessels disposition	Added understanding in complex vessels disposition
			Peroperative view
**SURGICAL GESTURE DECISION**			
Kiraly et al. (32)	Aortic arch obstruction	Arch repair modality?	Autologous flap repair without patch
**CATHETERIZATION PROCEDURES**			
Valverde et al. (18)	Aortic arch hypoplasia		Planning and training for the stenting
Olejnik et al. (17)	Complex CHD	Collateral arteries	Indication for cath embolization, for PB stenting
	*(Ambigous spatial anatomical relationship)*	PB morphology	

*TOF, Tetralogy of Fallot; ROLSCA, retro-oesophagian left sbuclaviar artery; TAPVR, total anomalous pulmonary veins return; 1V, one ventricle; DTGA, D transposition of great arteries; PAVSD, pulmonary atresia with ventricular septal defect; ROSC, retro-oesophagian subclaviar artery; DORV, double outlet right ventricle; HTx, heart transplantation; RPA, right pulmonary artery; MAPCA, major aortopulmonary collateral artery; CPB, cardiopulmonary bypass; PB, pulmonary branch*.

For these extracardiac structures, the better understanding of the anatomy and more convenient viewing of the structures facilitated the surgical planning and access to the vessels to be treated. When cardiopulmonary bypass was needed, some authors suggested that using 3D models could reduce the cross clamping time in complex cases but this as yet to be demonstrated Smith et al. (30).

**Figure 1:**
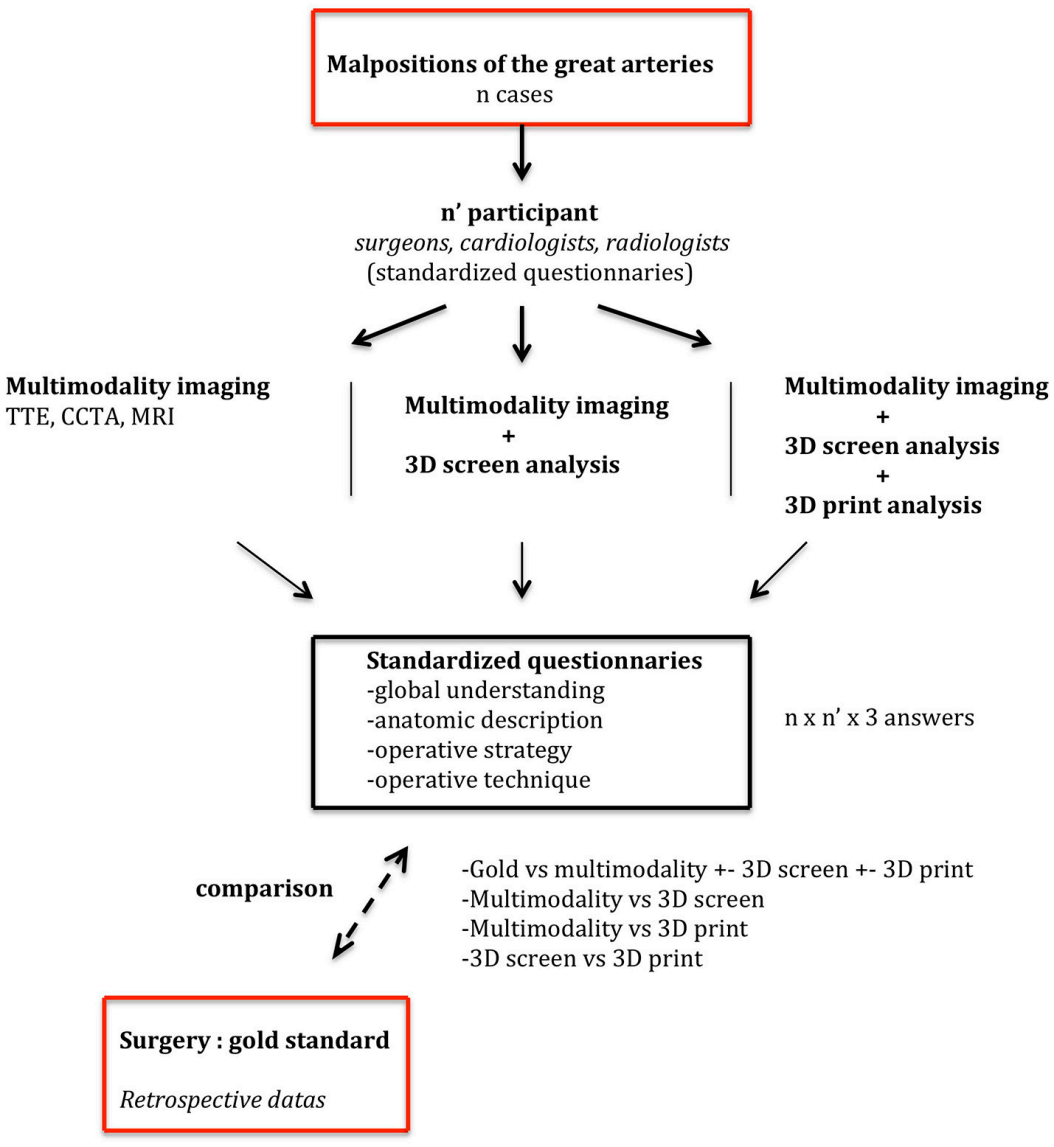
Design proposal for a retrospective study.

Along the same line, planning of the procedure could be accelerated in the cath lab using 3D models before pulmonary branches or aortic arch stenting, and to localize aorto-pulmonary collateral arteries to be embolized (Figure 1). Certainly, the combination of 3D models and fusion imaging in the cath lab should be developed to optimize percutaneous procedures. Still, the real benefit of these 3D models to guide procedures on extracardiac structures appears to be weak. Indeed, the clear demonstration of the anatomy is usually obtained with MRI and CT that offer 3D visualization of the vessels and clearly describe the relationships with adjacent thoracic structures. A potential development for the use of 3D models could be the geolocalization of the major aorto-pulmonary collateral arteries (MAPCAs) in pulmonary atresia with ventricular septal defect. Indeed, the cross clamping time might be excessively prolonged in this defect because the usual imaging modalities can describe the origin and course of the MAPCAs but their tracking might be complex.

The second group of case reports had the objective to analyze intracardiac structures, particularly the ventricular septal defects in complex malpositions of the great arteries to plan uni- or bi-ventricular repair as well as the technique to tunnelize the ventricular septal defect to one of the great vessels (see Supplementary Table 1) (9, 13, 17, 28, 29, 33–41)^.^. Indeed, the two main questions in these defects are the suitability for bi-ventricular repair and when apparently feasible, the choice of the surgical technique to create left and right outflow tracts. For this purpose, 3D models are complementary with echocardiography that is today the only modality that can describe the atrioventricular valves and their subvalvar apparatus adequately. The defects in which this type of approach could be useful are the double outlet right ventricles, the congenitally corrected transpositions of the great arteries and the criss-cross hearts. The simulation of the surgical procedure could also be better performed with the use of 3D models. Indeed, the planning of the VSD closure might be complex in these defects as well as the mental representation of the baffle between the left ventricle and the future aorta (see Supplementary Table 1).

### Case Series Using 3D Models for Planning of Surgery: Where Do We Stand?

Three series attempted to demonstrate the benefit of 3D models in planning surgical procedures in congenital heart diseases (Table 3). Ma et al. (16) prospectively analyzed 35 cases of tetralogy of Fallot with 3D printed models. The authors' main objective was to compare printed models with intra-operative findings. They showed that the 3D printed models were reliable to predict intra-operative findings. This study confirmed the reliability of 3D models but focused on a defect in which decision-making is consensually easy.

**Table 3:** Studies about benetif of 3D printing technology in complex CHD's surgical planning.

**References**	**Cases**	**CHD**	**Comparison**	**Result**	
Ma et al. (16)	35	TOF	Prospective monocentric description	**Better conditionnement and surgical planning**	
			3D printed model vs. peri-operative funding		
Valverde et al. (14)	40	19 DORV, 8 TGA, 4 UVH	Prospective multicentric case -crossover	**Surgery decision**	**Surgical planning**
		5 large VSD	Stage 1 strategy with usual exams	3/12 conservative -> surgery	12/28 no change
		1 LVOTO/CCTGA	Stage 2 strategy with 3D printed models	1/28 surgery -> conservative	15/28 change
		1 heterotaxy	Stage 3peri-operative strategy		1/28 no surgery
		3 criss cross heart	*Decision-making/strategy/global contribution*	Global contribution of 3D	21/40 : no impact of 3D
					19/40 : impact of 3D
Zhao et al. (19)	25	DORV	Measure GA and VSD	**0.094 accuracy**	
			Prospective comparison : 3D (8) vs. usual (17)		
			Time : *CBP (min), ACC (min)*	**Lower ventilation time** 56.43 ± 19.74; 96.76 ± 50.26 p = 0.040	
			*Ventilation (hours), ICU(days)*	**Lower ICU** 99.04 ± 16.13; 166.94 ± 90.30 p = 0.008	

*TOF, Tetralogy of Fallot; DORV, double outlet right ventricle; TGA, transposition of great arteries; UVH, univentricular heart; VSD, ventricular septal defect; LVOTO, left ventricular outflow tract obstruction; CCTGA, congenitally corrected transposition of great arteries; GA, great arteries; CPB, cardiopulmonary bypass; ACC, aortic cross clamping; ICU, intensive care unit*.

Valverde et al. (14) reported a multicentric prospective study using 3D models of complex CHD as double outlet right ventricle, complex transpositions of the great arteries, criss cross heart, and univentricular hearts. The design was a case-crossover method comparing the surgical indication using classical imaging modalities by a multidisciplinary team followed by the same process using 3D printed models, and finally compared to the surgical findings. In this study, the 3D models were considered helpful in optimizing the surgical planning in 50% of the cases. This interesting report does not give indication on the anatomical structures that were key to modify the decision and grouped a large variety of anatomies. It remains therefore difficult to foresee the indications for the use of 3D models for this purpose.

The third study by Zhao et al. (19) evaluated the benefit of the use of 3D models in 25 double outlet right ventricles. The end-point was clinically meaningful as they showed that ventilation time and ICU stay were shorter in the group analyzed with 3D models suggesting a shorter time in the operating room and/or a more appropriate strategy. This study clearly indicates that 3D models could help optimizing surgical planning in complex CHD. The limitation was essentially related to the unclear description of cases selection and randomization.

## Discussion and Conclusion

### How to Design a Study to Demonstrate the Benefit of 3D Models in the Surgical Approach of Complex CHD?

To be convincing, a clinical study using 3D models should have a clinically meaningful end-point. This type of hard end-point could only be obtained through a prospective study that will compare a homogeneous group of complex CHD. Hard end-points that could be obtained in the operating room (cross-clamping time) are questionable as the variety of procedures and surgical skills are difficult to control. In addition, the surgical strategy can certainly not be completely planned due to the diversity of the defects and associated anomalies. Post-operative end-points such as ICU stay may also be patient dependent and therefore randomization and stratification strategies would lead to an excessive number of patients to be included. We estimate that the first step to demonstrate the convenience and usefulness of 3D models in complex CHD should be a retrospective study aiming to compare the added value of 3D models to usual multimodality imaging to plan surgical repair. Indeed, the group of malposition of the great arteries is the most challenging group of CHD and the different surgical techniques available for biventricular repair require a precise evaluation of intracardiac anatomy. The primary end-point of this type of study could be the proportion of predicted surgical repair using conventional imaging modalities without or with the 3D model. Secondary or exploratory end-points that would be of interest are the additional information obtained from 3D models and potentially the optimization of surgical strategy. Cardiac surgeons, congenital cardiologists and radiologists should be involved and blinded of the final procedure (Figure 1).

This type of retrospective design might prove the complementary value of 3D models in planning surgical repair in this group of patients. If positive, a prospective design with hard end-points could be built to attempt to show a clinical benefit in a larger population.

An immediate and friendly use of these models would be for teaching purposes. Indeed, organizing hands-on session with specimen is always a complex process and this is possible only in institution who have a collection of specimens. The presence of a trained anatomist is mandatory and the risk is to damage the specimens during this type of teaching session. 3D printing is an elegant way to transmit anatomical knowledge in complex CHD. The 3D models can be obtained from living patients but they can also be obtained from high resolution CT of specimen. We are currently scanning our collection to build a virtual and printed new collection of CHD specimen with the objectives to preserve it and to teach CHD anatomy.

### Conclusion and Perspectives

3D modeling and 3D printing technologies are reliable and suitable for the analysis of congenital heart diseases. Hitherto, however, 3D has its own limits and potential bias, related to the part of subjectivity in the process of modelizing CHD. Today, it remains an additional tool in the decision making for complex CHD. There is a need to improve the process for obtaining and manipulating 3D models in the daily clinical practice. The final step would be to use 3D models as a summary of the diagnosis in a given patient and as a reliable tool to decide which treatment has to be applied. This evidence-based information should be obtained through studies with clinically meaningful end-points.

## Author Contributions

CB, MH, and DB conceived of the presented idea. CB and DB performed the research and wrote the review together. CB and MH performed the printing and the surgery simulation illustrated in Figure 1.

### Conflict of Interest Statement

The authors declare that the research was conducted in the absence of any commercial or financial relationships that could be construed as a potential conflict of interest.

## Abbreviations

CHD: Congenital Heart Disease
DICOM: Digital and Imaging Communication in Medicine
CT: Computed Tomography
MRI: Magnetic Resonance Imaging
TTE: Trans Thoracic Echocardiography
STL: Standard Tessellation Language
FDM: Fused Deposition Modeling
MAPCA: Major Aorto-Pulmonary Collateral Artery
ICU: Intensive Care Unit.
