# Supplementary material for: 3D-Printed Models for Surgical Planning in Complex Congenital Heart Diseases: A Systematic Review
Source: Front Pediatr. 2019 Feb 11;7:23. doi: 10.3389/fped.2019.00023 (PMC6378296; doi:10.3389/fped.2019.00023)
Supplement: Supplementary file 1 [file Table_1.docx]

| **Authors** | | **CHD** | | | | **Problematic** | **Benefit of 3D printed models** | |  |  |
| --- | --- | --- | --- | --- | --- | --- | --- | --- | --- | --- |
| Mottl-link et al^33^ | | V-A discordance with AOCA | | | Anatomic complexity of intracardiac connection | Intra-operative view | | |  |  |
| Farooqi et al^34^ | | DORV | | | Uni or bi ventricular? | Change strategy to bi ventricular repair | | |  |  |
| Bathla et al^35^ | | DORV | | | VSD relationship with GA and closability | Feasability to close the VSD via TV | | |  |  |
| Farooqi et al^36^ | | DORV | | | VSD relationship with GA and closability | Double switch decision | | |  |  |
| Valverde et al^37^ | | TGA-VSD-PS | | | Surgical gesture | Help Bex Nikaidoh decision | | |  |  |
| Olejnik et al^17^ | | Complex CHD | | | Surgery indication? | Help Indication for surgery, for biV repair | | |  |  |
|  | | *(ambigous spatial anatomical relationship)* | | | *Surgical modality?* |  | | |  |  |
|  | |  | | |  |  | | |  |  |
| Kappanayil et al^38^ | | *1 DORV, 1 AV discordance, 1 criss-cross-DORV* | | | Unresolved cases (surgery indication | Modify pre-operative surgical decision (impropbabilty to biV repair) | | |  |  |
|  | | *1 criss-cross-vsd, 1 multiple VSD* | | | or modalities) | Modify surgical approach and gesture : upgrade operative view | | |  |  |
|  | |  | | |  | Understand LV-OT tunnel, approach a VSD closure  Approach a biV repair by Rastelli | | |  |  |
|  | |  | | |  |  | | |  |  |
| Schmauss et al^29^ | | VSD,2 AO stenosis, RV tumor | | | Surgical approach? | Perioperative orientation | | |  |  |
|  | |  | | |  |  | | |  |  |
|  | |  | | |  |  | | |  |  |
| Bhatla et al^39^ | | 3 DORV, 3 complex multiple VSD | | | VSD relationship with GA and closability | Anatomy : relation GA-VSD, conal septum, AV valve attach | | |  |  |
|  | |  | | |  | Planning : Residual VSD closability, complex VSD closure | | |  |  |
|  | |  | | |  |  | | |  |  |
|  | |  | | |  |  | | |  |  |
|  | |  | | |  |  | | |  |  |
| *Riesenkampff et al^40^* | | Complex CHD | | | VSD morpho and relation to OT | Additional support on surgical decision and planning | | |  |  |
|  | | with equivocal surgery decision | | |  | 3D printed models agree with io findings | | |  |  |
|  | |  | | |  |  | | |  |  |
| Anwar et al^28^ | | TOF, 3 DORV, superposed V, DTGA-Senning | | | Complex anatomy and spatial relationship | Surgical planning and surgical approach | | |  |  |
|  | |  | | |  | Guide a VSD baffle pathway or an interatrial baffle. | | |  |  |
|  | |  | | |  |  | | |  |  |
| Yoo et al^9^ | | DORV | | | Complex anatomy and equivocal perception | Procedure prediction | | |  |  |
|  | |  | | | misinterpretation and misunderstanding | Surgical tailoring for specific cases | | |  |  |
|  | |  | | |  |  | | |  |  |
| *Garekar et al^41^* | | DORV with balanced V | | | Is 3D printed model usefull? | 3D printed model often give extra-information | | |  |  |
|  | |  | | | Score : 3D model vs MR/CT or vs TTE or vs io finding | for anatomy understanding, surgical prediction and decision-making | | |  |  |
|  | |  | | | 11 questions' scale on anatomy |  | | |  |  |
|  | |  | | |  |  | | |  |  |
| *Lau et al^13^* | | DORV with sub-aortic VSD | | | Is 3D printed model usefull? | | Surgical planning 4/2 Simulation 3/6 | | |  |
|  | | |  | 2 radiologists, 2 cardiologists, 2 surgeons | | | Satisfaction score 8,4/10 | | | |

Supplementary Table S1 : case reports and single-center experience studies about benefits of 3D-printed models in congenital heart diseases ‘ surgical planning considering intra-cardiac anatomy.

VA : ventricular-artery, AOCA : anomalous origin of coronary artery, DORV : double outlet right ventricle, TGA : transposition of great arteries, VSD : ventricular septal defect, PS : pulmonary stenosis, AO : aorta RV : right ventricle, TOF : Tetralogy of Fallot, OT : outflow tract, IO : intra-operative, TV : tricuspid valve, GA : great arteries, LVOT : left ventricular outflow tract.
